# The Value of Co-operation in the Sanitary Control of Our Periodic Epizoötics of Anthrax1Read before the Louisiana State Medical Society, Shreveport, June, 1902.

**Published:** 1902-08

**Authors:** W. H. Dalrymple

**Affiliations:** Louisiana State University, Baton Rouge, La.


### THE VALUE OF CO-OPERATION IN THE SANITARY CONTROL OF OUR PERIODIC EPIZOOTICS OF ANTHRAX.[1]

By W. H. Dalrymple, M.R.C.V.S.,
LOUISIANA STATE UNIVERSITY, BATON ROUGE, LA.

I can assure you I esteem it a great privilege and honor to have been invited to present a paper before the State Medical Society of Louisiana.

The study of pathology covers a very wide field, and is not, by any means, confined to pathological conditions affecting only the genus homo, but is similar, whether it is applied to investigations affecting man, the lower animals, or even plants; and the same may be said of hygiene and of sanitary science in general, especially as we are now aware, through the indefatigable efforts of the workers in the great field of bacteriology, that disease everywhere depends so extensively upon the presence of the ubiquitous microbe.

Fortunately for us, the pathological forms are in the minority, although we are inclined, from our familiarity with them, I presume, to think of bacteria as disease-producers only. Such an impression, however, is altogether erroneous, as the great majority of these lowly forms of life are of great economic value, and, therefore, helpful to man in many spheres of life.

[1] Read before the Louisiana State Medical Society, Shreveport, June, 1902.

It is but a year ago that the agriculturist was wont to look upon his soil as a mass of dead, inert matter, but which, by some unaccountable and mysterious agency, became fertile through the application of artificial or other manures. To-day the mystery is all cleared away, and he now knows that what he once thought to be sterile earth is one immense laboratory of germ-life busily engaged in the process of nitrification, manufacturing the insoluble fertilizing materials into soluble, and, therefore, available plant-food to be taken up by the delicate rootlets for the tissue-building of the plant.

Only a few years since two German scientists (Wilfarth and Nellreigel) elucidated the problem of the enrichment of soil consequent upon the growing of leguminous crops, which previously had been clothed in mystery. Following up their investigations they discovered that the various plants belonging to the natural order Leguminosæ had on their tiny rootlets, and root-hairs, minute nodules or tubercles, and that these enlargements contained within them great numbers of micro-organisms having the power of fixing the free nitrogen of the atmosphere, the most costly of fertilizing elements, and elaborating it for the use and benefit both of the plant and of the soil.

The great dairy industry has been the beneficiary of a discovery of germ-life where it was, perhaps, least suspected. Not so long ago that fragrant and delicious bouquet which we all desire so much in our butter was found to be a product resulting from the labors of a flavor-producing bacterium, the culture from which can now be purchased as a commercial commodity, and is within the reach of all engaged in this industry; and we are all familiar, of course, with the great value to various industries that the discovery of the different fermentations has been. But I have just alluded to one or two of the more recent investigations into the life-history of some of the economic bacteria to emphasize the fact that some forms—in fact, the much larger number—are not only beneficial, but necessary in this work-a-day world of ours.

Unfortunately, however, although few in number, the pathogenic forms are amply sufficient to carry destruction and devastation in their wake to the various forms of animal life with which we have to deal.

Of the different classes of pathogenic organisms, those which play the rôle of etiological factors in the production of transmissible and intercommunicable diseases may be said to be the most important from the standpoint of the pathologist, whether special or comparative, among which may be mentioned the bacillus anthracis, the bacillus tuberculosis, the bacillus mallei, the bacillus tetani, etc., all being spore-bearing, and, therefore, capable of a much more extended existence and a greater tenacity than are those that do not have a spore-bearing stage in their life cycle.

Anthrax, the disease which is to occupy our attention for a short while on this occasion, is produced by one of the spore-bearing forms, and it is to the tenacity or vitality of the spores, outside of the animal body, is due the permanent infection of certain localities consequent upon the careless and improper disposal of anthrax cadavers, which has, unfortunately, been the rule rather than the exception, in this and some of the other States for many years. This fact will be more readily appreciated when it is known that anthrax is a true septicæmia; that is to say, the bacilli are always found in immense numbers in the blood of the charbonous victim, at all events, immediately preceding and after death. Hence the extreme virulence of the blood and tissues of the anthrax carcass.

The fact that this disease has been permitted to exist to such a length of time in our midst, and with such periodic fatal results, has, I think, been due almost solely to the general lack of information relative to its true nature. Scientific progress, therefore, in endeavoring to ameliorate conditions, has been somewhat slow, since the struggle has been against the enormous odds of incredulity, indifference, and ignorance; and, like all great reform measures, satisfactory results depend largely upon the education of public opinion.

It is not altogether astonishing, however, that the importance of such a contagious and fatal malady to man and beast should not have received more attention at the hands of the pathologist in our midst, or more appreciation by the laity, when we find such an eminent authority as Surgeon-General Sternberg stating, in his voluminous *Manual of Bacteriology*, that "anthrax does not prevail in the United States," a statement, I need hardly say, for which there is absolutely no foundation in fact.

Before approaching the more specific part of my subject I have thought it might be both interesting and acceptable to allude briefly to the historical, bacteriological, and hygienic (including sanitary) divisions, in order that what application I may make may be the more intelligible and pointed.

Throughout the civilized world this disease is known by various names and synonyms. By the Latin term anthrax it is known to the English-speaking medical world, although it has also received the names wool-sorters' disease, disease of rag-pickers, malignant pustule, carbuncular disease, splenic apoplexy, and splenic fever. It is most familiarly known to us by its French synomyn, charbon. The Germans speak of it as miltzbrand, or disease of the spleen. In Italy, carbonchio is given to it; while the Russians allude to it as jaswa, or siberskaji, jaswa, etc.

It is possible that anthrax is the most contagious and the oldest disease of animals known to medical science. It is said that the "sixth Egyptian plague," spoken of by Moses in his second book, was this very malady; and in his third book he indicates the transmission of anthrax to man by the intermediation of soiled clothing. The epizootic described by Homer in the first book of the *Iliad*, and which affected man, the mule, and the dog, was probably no other than anthrax. In the ninth book of his *Metamorphoses*, Ovid gives an exact description of anthrax epizootic.

Plutarch has given the history of an outbreak which existed in Rome about the year 740 before the Christian era. Dionysius, of Halicarnassus, 488, and Livius, 425, B.C., have mentioned examples of anthrax affections which exist first in animals living upon pastures, then in such as were kept in stables, in animals destined for sacrifices, in priests, shepherds and farmers, and lastly upon the whole population. So much for ancient history.

Coming down to a later date, we find that in 1617 the disease was prevalent, and of such a fatal nature, in the neighborhood of Naples, Italy, that over 60,000 people perished through eating the flesh of animals that had died of anthrax. In 1731 it made its appearance in several provinces of France. The years 1757, 1763, 1775, 1779, 1780 and 1800 were signalized by the charbonous malady, which extended nearly all over France and affected all the domestic animals. From 1800 to 1846 many outbreaks were observed, generally in the hottest months. In Russia, during 1864, in the five governments of St. Petersburg, Novgorod, Olentz, Twer, and Jaroslaw, over 10,000 horses and nearly 1000 persons perished from the disease. I might refer to records of devastation caused by anthrax in still other countries, but it seems unnecessary to go beyond the limits of our State for authentic information regarding the ravages of charbon, both in lower animals and, unfortunately, in many human beings as well.

In making some investigations, two or three summers ago, into the history of anthrax in Louisiana, I found that outbreaks were known as far back as some of the oldest citizens could recollect, notwithstanding the statement of General Sternberg to the contrary; and it is probable the contagion has existed and outbreaks of the disease have appeared periodically for generations before its true etiological factor was known or even suspected. Consequently sanitary measures were not thought of to ameliorate existing conditions; the contagion was permitted to spread through the medium of various agencies, and permanent infection given a foothold in those sections most favorable to the development of the specific organism.

Anthrax, then, is a specific disease, and can be produced only through the medium of its own specific germ, the bacillus anthracis, or, as I understand the more recent nomenclature of Miguel gives it, the bacterium anthracis, although it was not until 1845 that charbon was believed to be contagious, when in that year Gerlach demonstrated it experimentally. In 1855 Pollender, of Wipperfurth, announced that in 1849 he had found in the blood of cattle afflicted with anthrax a considerable quantity of fine little sticks. These were also seen by Duvaine, of Paris, in 1850, and by Branell, of Dorpat, in 1857. This last investigator based his diagnosis of anthrax upon the presence of these little sticks in the blood, but he denied their disease-producing properties.

It was Duvaine, who, in 1863, recognized that these elements were bacteria, and that they constituted the specific agents of charbon. Cohn was the first who considered the little sticks bacilli and suspected their sporulation. Delafond was the first who tried the cultivation of anthrax rods, and by exposing virulent blood to air he said that these little sticks became lengthened. The renowned Koch, however, threw more light upon the development of spores and their transformation into bacilli.

A great deal of interest must ever circle around this charbon bacillus when we consider for a moment that the great and invaluable science of bacteriology has been developed through the identification of this pathogenic organism.

In order to have a proper appreciation of the hygienic treatment of anthrax we ought to know something of its bacteriology—the various channels by which the organism gains access to the animal economy and the numerous media of transmission.

There are two great classes of bacteria; those requiring oxygen for their existence being termed aërobes; while the anaërobic germs find in the organism deprived of oxygenated blood the most favorable conditions for their multiplication.

It is to the first, or aërobic class, that the germ of anthrax belongs, and must have oxygen for its life and development; and right at this point a very important sanitary fact seems to "fit in."

When an animal succumbs to anthrax, the vital food, so to speak, of the germ is cut off; and if the body is kept intact, and no blood is permitted to escape from the natural or other openings, it will be found on microscopic examination, twenty-four or thirty-six hours after death, that it will be difficult to discover an anthrax organism, they having evidently degenerated and died for want of oxygen, and become the prey of the putrefactive bacteria. If, then, the precaution is taken to prevent the escape of this germ-laden blood, and the carcass is immediately incinerated, the danger of spread or contamination from it is reduced to a minimum.

But, on the other hand, should blood be allowed to escape from the body through any channel whatever, the atmospheric oxygen is sufficient to revivify the bacilli it contains; and by a dying effort of nature to reproduce themselves, as it were, they are enabled to sporulate, the surroundings are contaminated with the virulent blood, causing no end of future trouble and probably permanent infection in the immediate locality.

In the blood of the living animal the anthrax organism exists only in the bacillus or rod-like form, and multiplies by fission or segmentation. Spores are not found in the vital fluid during life. Outside of the body we do not find bacilli, but only the spore form; and these, being much more resistant to external destructive influences, are capable of living, probably indefinitely, until they are taken into the system and there develop into bacilli. This is the generally accepted life-history, but it is possible that conditions may sometimes arise sufficiently favorable, outside the body, for the various phases in the life-cycle of the germ to be completed. The former, however, must be the most frequent, as examinations of anthrax-infected materials—with the exception of fresh blood—generally reveal the organism in the spore stage.

There are three recognized ways by which the germ of anthrax may gain access into the animal body, viz., by the alimentary tract, by the skin, and by the lungs; but it is rare for the disease to be transmitted directly from a diseased to a healthy animal.

Intestinal or internal anthrax is usually produced by spores which are ingested with contaminated food and water.

This mode of infection is frequent with us in the initial stage, and during the course of an epizootic, through previous infection in the first instance, and continued infection in the second, attributable in each case to the want of sanitary care in the disposal of animals that have died of the disease, their discharges having infected the surroundings grazed over by healthy stock. The virulent elements, after ingestion, in such cases gain access to the circulation through the delicate intestinal wall.

Cutaneous or skin infection produces the form known as external anthrax, carbuncular disease, malignant pustule, etc.

This variety is brought about by some method of external inoculation. In the lower Mississippi Valley, at least, the numerous varieties of flies belonging to the order "Tabanidæ," or horse-flies, are, in my opinion, more responsible than any other cause of transmission in the lower animals, when they are first provided with a source, such as charbonous blood, from which to obtain the virus. Of course, you are all familiar with the external form of anthrax in the human being, and the various media of transmission, some of which I will refer to a little later.

The third channel of infection is the respiratory tract, the spores, in a desiccated condition, coming in contact, through inhalation, with the mucous membrane of the delicate air-tubes and acini in the lungs. This form is known as pulmonary anthrax, woolsorters' disease, disease of rag-pickers, etc., and it has been clearly established that the mode of entrance of the spores is through the respiratory organs.

Although the human subject, and especially those engaged in the business of wool-sorting, rag-picking, mattress-making, etc., may frequently contract anthrax in this way, I am inclined to think that in the lower animals it is by no means so common as by ingestion and inoculation. In fact, I should consider it rare, unless in infected areas during very dry, dusty weather, the spores in a desiccated state might be carried by the wind and inhaled, or taken in through the same channel from dusty, contaminated hay or other feeding material which has grown on an infected soil and containing spores of anthrax.

Before touching upon the various transmitting agencies it may be asserted that, as the organisms of anthrax are found in great numbers in the blood of the cadaver, this, as well as all other tissues supplied by it, is virulent, and, therefore, capable of transmitting the contagion when brought in contact with any medium by which it may be conveyed.

In the case of human beings, the contraction of anthrax from the ingestion of charbonous flesh, insufficiently cooked to destroy the spores, although at one time apparently common, can now be looked upon as of somewhat rare occurrence, I think, owing to more modern methods in the preparation of meats used for human food.

The cutaneous and pulmonary forms of anthrax are of most frequent occurrence in man at the present time, and of the former there are some interesting records. For example, I have observed in an excerpt from the *Medical Press and Circular* that an English physician, residing in the island of Cyprus, encountered some remarkable cases of disease transmitted in the sting of the solitary ant. In one instance a woman was stung during sleep by one of these insects, and the wound showed active signs of anthrax infection. It was found upon investigation that in a field adjoining her cottage there was a dead sheep which had laid there for a week after having died of that disease. The article adds: "Here, then, is yet another insect to be added to the growing list of those that, while not dangerous in themselves, are capable of great mischief, owing to their transmission of malignant bacilli."

A few years ago an employé of the London general postoffice succumbed to anthrax infection by being inoculated with a piece of leather out of which he had been manufacturing hinges for a box in which to carry packages. It has been clearly demonstrated by actual test that the modern process of tanning is not sufficient to kill the spores that may be present in the blood of hides of charbonous animals. Hence the leather manipulated by this man must evidently have contained virulent anthrax infection.

Only last year I read of a case in an English medical journal where some boys became infected through taking to pieces the machinery connected with a carding mill. It not infrequently happens that the skins and wool of sheep that have died of anthrax find their way to the market. The wool may very easily become contaminated with charbonous blood, and, in the process of carding, the spores get on to the parts of the machinery. This, no doubt, was the manner in which the infection was transmitted to the unfortunate boys, who, at the same time had received abrasions of the skin of the hands.

The skinning of charbonous cadavers is, perhaps, the most frequent way in which external infection is produced in man; and right at this point I would like to emphasize the danger to human life, in the first place, and of spreading the contagion, in the second, by such procedure, which cannot be too severely deprecated.

There is hardly a year in which there are not cases of cutaneous anthrax among employés in the various tanning districts throughout the country by inoculation from the virulent blood of hides imported from countries in which the disease is prevalent, and the refuse from such tanneries being carried by drains and streams frequently finds its way on to meadows and causes extensive outbreaks of anthrax in cattle and other herbivorous animals grazing upon them. This mode of bringing anthrax into the United States has caused the customs department to require a consular certificate with all imports of hides, of one kind and another, from various countries in which infectious and contagious diseases, such as anthrax, etc., prevail. Such certificate is required to show that all imports of hides are either dry-salted, arsenic or lime-cured, and thoroughly disinfected according to the sulphur formula prescribed by the secretary of the treasury. Exceptions are made in the case of hides shipped from Great Britain, Norway, and Sweden abattoirs, for in these countries only cattle absolutely free from all diseases may be slaughtered.

The pulmonary form (wool-sorters' disease) in man is introduced through wool and horse-hair, in a similar manner to that above alluded to in the case of the boys employed in the carding mill ; but, instead of external infection, the dried spores, mingled with dust and floating in the atmosphere of the rooms or workshops, are inhaled and find lodgement in the respiratory organs.

Coming to the lower animals and the different ways in which anthrax infection is conveyed, I will first consider feeding materials.

When herbivorous animals graze over pastures—and more especially if the grass be short—that have been soiled by the blood or other discharges from exposed charbonous carcasses, and the climatic and meteorologic conditions be favorable for the development of germ-life, they contract the disease through ingestion of the spores, producing internal or intestinal anthrax or charbon.

This is quite frequent in this State, more particularly during the early part of the season. But it is also possible, and in fact often happens, that anthrax is introduced, where it had not previously existed, through the medium of hay, grain, and manufactured foodstuffs that had been raised on infected ground.

I have had personal experience of at least two outbreaks brought in this way. The first was in a valuable herd of Jersey cattle in the State of Georgia, where the disease had not previously been known. The food in use was one of the "commercial feeds," made up principally of the offal of different cereals, and sold as cattle feed. Some of the grains had evidently been raised on an infected area, and contained anthrax spores. No animals on the farm died except those fed this food; and one or two others succumbed, belonging to another owner who had borrowed some food for the purpose of trying it. It proved an expensive loan.

The other outbreak occurred in this State about seven years ago, and the fatalities amounted to some twenty head of sugar mules.

The vehicle in this instance was bran from one of the cereals, which, like the previous case, must have been raised in an infected district. A cessation of the disease followed the stoppage of the use of this food material, but which, after again resorting to its use, on account of the owner doubting its virulent character, destroyed several more animals. After total disuse of the bran there were no more cases. Anthrax had not been known to exist on this place before, or, at all events, within the recollection of the oldest inhabitant.

A somewhat celebrated lawsuit occurred only last year over an outbreak of anthrax on a farm in the north of England, thought to be introduced in some oil-cake for feeding purposes. The cake was examined, microscopically and bacteriologically, by a number of eminent British pathologists (among them Prof. John McFadyean, of London, one of the ablest in Europe, and a gentleman whom you may remember as replying to the famous paper of Dr. Koch at the last Congress of Tuberculosis held in London, and whom, I trust, I may be pardoned for referring to as a personal friend of my own), and was found to be infected with anthrax spores.

Water is another vehicle by which the contagion may be carried. This can readily be inferred from my previous allusion to infection being conveyed by running water from tanneries. Rains washing the virulent germ-laden blood from the surroundings of the exposed charbonous body can easily carry the contagion into streams, etc., and, on falling or receding, infection is left along their banks where animals graze, which may be quite a distance from the primarily infected locality.

Our carrion birds, as the buzzard and carrion crow, are, in my opinion, very important factors in spreading infection. For, after contaminating their feet with virulent blood from a charbonous victim, they may infect pastures wherever they alight, which may be miles from where they obtain the virus.

Our carnivorous and omnivorous animals, also, play quite a prominent part as disseminators of the contagion. After feeding upon the charbonous flesh, they may live long enough to travel great distances, infecting as they go, before they, too, succumb to become fresh foci of the disease and keep up its spread.

Among the agencies responsible for the transmission of the disease in its carbuncular or external form, however, the numerous varieties of the tabanidæ, or horse-flies, are, in our section of the country, undoubtedly the most potent. Of these there are several hundred, and the conditions seem to prevail which permit of their successful development and multiplication.

Porchinski, a Russian entomologist who has made a study of the life-history and habits of this hitherto somewhat neglected order of insects, states that: "Water and arboreal plants are the chief conditions of the existence and multiplication of the family to which horse- or gad-flies belong; and where these conditions are absent, no tabanidæ are observed." Now, all such favorable conditions we possess in abundance in those sections of the State which suffer from anthrax.

It has been a matter of general observation that outbreaks of this disease in epizootic form usually succeed periods of protracted drought in summer and after the breaking of such drought by the first few showers of rain. On the other hand, the disease rarely occurs over an extended area, and if at all in only sporadic or enzootic form during seasons in which we have frequent and copious precipitation. This may be accounted for, first of all, by the fact that a lengthened dry spell of weather favors the development and multiplication of greater numbers of horse-flies, which would be destroyed in the oval or larval stages by incessant heavy rains during these more delicate stages of the insect's life.

Then, again, the moisture from the showers following the dry weather, combined with the natural heat of our summers, brings about conditions favorable to the development of the latent bacterial life already in existence in infected localities.

When heavy and frequent rains continue during our summers we seem to have fewer of these flies, for the reason, no doubt, just stated, and it seems reasonable to presume that a great deal of infection is washed from the surface of the ground and of the vegetation, and carried away by running water, which, unfortunately, however, becomes a menace to territory below, or through which such contaminated water passes.

Given, then even one charbonous carcass, with its infected blood and other tissues, left carelessly exposed on the surface of the ground, and a favorable season for the propagation and development of these blood-sucking flies, which will attack the animal even after death while the germ-laden blood is yet warm, and the merest tyro can conceive how hundreds or perhaps thousands of inoculations can be produced in healthy animals over quite a wide extent of territory. But, get rid of the anthrax-cadaver by incineration, if possible, and all transmitting agencies are deprived of the source from which the virus can be conveyed.

This is nothing more than a simple yet most important sanitary proposition, which if it had been more strictly carried out in previous years would have vastly restricted the area of infection in the State and been the means of a saving to our citizens of probably millions of dollars beside the lives of many individuals who have become victims to the disease through inoculation.

But although it may be improbable or even impossible to be able to rid the State of contagion, and especially from those localities which may be termed infected districts, that have been infected and reinfected from time immemorial, through want of a correct knowledge of sanitary science as applied to anthrax, it is possible to greatly limit the further spread of the contagion by the adoption of the proper sanitary measures in the future, and by immunizing our animals against fatal attacks of the disease at least. These include the careful and proper disposal of all charbonous carcasses, the thorough disinfection of localities and articles liable to have become infected, and preventive inoculation with prepared lymph to render our animals refractory.

This now brings us to the subject of artificial immunization, which I feel sure will be of some interest to those of you who may not have given to the matter any very serious consideration up to the present time.

It may be said that the most noteworthy application of artificially prepared living vaccines to the protection of animals against infection is seen in connection with this disease—anthrax.

The first preventive inoculations were made by Toussaint. This investigator used defibrinated blood of anthrax heated for ten to fifteen minutes at a temperature of 50° to 55° C. He did not secure, however, the true mechanism of immunity which he thought he had thus produced. The virulence of charbon cultures is very stable on account of the spores, which are little subject to change, and when it is desired to attenuate these cultures it is necessary to begin by preventing the formation of spores. Pasteur succeeded in this by cultivating anthrax bacilli at the temperature of 42° to 43° C., in the presence of oxygen. Multiplication of the bacilli still continues, but spores are no longer formed. The bacilli which have become asporogenous at the above-named temperature then lose their virulent properties, retaining only those of an ordinary saprogenic organism, and finally lose all their vitality.

What is in use now as "anthrax vaccine" is a lymph containing cultures of these attenuated asporogenous bacilli. This material is generally used in two strengths. The preparation of the first virus, which is the weaker, requires twenty-four days' exposure; that of the second twelve days; and they are inoculated at an interval of ten to twelve days apart; and immunity is conferred by this material for a period of about twelve months. I will not tax your patience by recounting the successes obtained in other countries by this immunizing method.

We have sufficient evidence in this State of its efficacy from the mere fact that previous to six years or so ago its use was almost totally unknown, and to-day probably 40,000 to 50,000 doses are employed annually throughout the State.

For the most satisfactory results the vaccine used must be thoroughly reliable; the operation performed early in the season; the syringe and needles and the skin at the point of puncture carefully sterilized; the animal kept from exposure to infection either from flies or from grazing on suspected pastures during the interval between the first and second or for the prescribed time (some ten to fourteen days) after the administration of the second lymph. On the other hand, if all the necessary precautions are not observed in dealing with such a dangerous and fatal malady, it is impossible to expect anything short of failure or at least indifferent success.

I am afraid, gentlemen, you will think the application of my topic has required a very lengthy preface, the greater portion of which might have been omitted; but I felt that the subject was of sufficient importance to warrant a somewhat extended résumé in order that the application might be the more intelligible, and capable of carrying greater conviction with it.

We are face to face with a condition of a very serious nature, menacing the lives, not only of our animals, but of our human population, and which I think calls for greater co-operative action. Living in the midst of such periodic fatal epizootics of anthrax, we do not seem to realize the gravity of the situation as do our Northern neighbors, who are inclined to look upon Louisiana and the lower Mississippi Valley in general, in this connection, with a feeling of almost perfect dread. A member of the American Veterinary Medical Association who visited this State during the prevalence of anthrax last year stated before the annual meeting of that Association, in Atlantic City, N. J., last September, that "we Northern men have not the faintest conception of the condition prevailing on account of anthrax that the veterinarians of Louisiana and Mississippi have had to contend with."

Now, the thought has frequently occurred to me when visiting outbreaks of anthrax throughout the country parishes that if practising physicians in those districts visited periodically by this disease would impart some of their knowledge of sanitary science to their clients, over whom they exert the usual wholesome influence of the family physician, telling them of the danger to be apprehended, and suggesting to them or directing them as to the absolute necessity for the exercise of strict sanitation, such as the complete destruction of carcasses and thorough disinfection, it would have a telling effect in preventing the spread of contagion in charbonous times. I do know of country practitioners who utilize their information in the education of their clients along such lines, and with splendid results. But I have heard of others who, on being asked for information, because the victim of anthrax happened to be a mule or a cow, exclaimed, with an air of wounded dignity: "I'm no mule or cow doctor, and don't know anything about it." The result of this has often been that some illiterate person, without any sanitary knowledge whatever, has been called in, and the contagion permitted to spread broadcast. This is surely not the spirit of the true pathologist. He looks upon disease as such, and does not consider the subject that accidentally has become the victim of it. Besides, in the case of the disease under consideration, its control and suppression should enlist the serious attention of the medical sanitarian, it being communicable to and extremely fatal in the human family, as well as the lower animals, as records of the past clearly show. It is well that it should be borne in mind, also, that the magnificent strides medical science has taken, and the exalted pinnacle to which it has attained in recent years, has been made possible largely through the untiring efforts of eminent research-workers in the great fields of comparative anatomy, physiology, pathology, and therapeutics.

It must not be inferred for a moment, however, that I am trying to suggest the necessity for physicians treating cases of charbon in animals. That is by no means what I am aiming at. My object is a purely educational one, and it can be accomplished at the expense of a very limited amount of effort. I am firmly of the opinion that in charbonous sections of the State, and where no educated and competent veterinarians are located, such sections being quite numerous, if, as I have previously suggested, the physician would, through his knowledge of contagious diseases, especially charbon, and of sanitary science, which is the same whether applied in the case of the higher or the lower animal, exert the influence he possesses in enlightening his client, the owner, as to the best measure to adopt to circumscribe contagion and eradicate the disease, the beneficent results accruing from such a source would, in a few years, I feel convinced, exceed our most sanguine expectations.

As I stated at the outset of my paper, pathology is a broad subject and not confined to the study of disease in any one species. The investigation of those maladies that are intercommunicable should be of as much interest to the human as to the comparative pathologist, so much depending upon their control and extermination, in the interest of the health of the human as well as that of the domestic animals.

The control of anthrax is a matter which, in reality, concerns the public health as well as the pockets of our stock owners, and any measures calculated to bring about amelioration of existing conditions would not only aid in the conservation of the public health, but of the public wealth also. And I feel confident that a little more co-operation all over the State in the sanitary control of our periodic epizootics would tend very materially toward the more speedy accomplishment of the end in view.

